# Preoperative treatment with metyrapone in patients with Cushing’s syndrome due to adrenal adenoma: a pilot prospective study

**DOI:** 10.1530/EC-18-0400

**Published:** 2018-09-20

**Authors:** Soraya Puglisi, Paola Perotti, Mattia Barbot, Paolo Cosio, Carla Scaroni, Antonio Stigliano, Pina Lardo, Valentina Morelli, Elisa Polledri, Iacopo Chiodini, Giuseppe Reimondo, Anna Pia, Massimo Terzolo

**Affiliations:** 1 Internal Medicine 1Department of Clinical and Biological Sciences, University of Turin, Turin, Italy; 2 Endocrinology UnitDepartment of Medicine DIMED, University of Padua, Padua, Italy; 3 Endocrinology UnitDepartment of Clinical and Molecular Medicine, Sant’Andrea Hospital University of Rome, Rome, Italy; 4 Endocrinology UnitDepartment of Clinical Sciences and Community Health, University of Milan and Fondazione IRCCS Ca’ Granda, Ospedale Maggiore Policlinico, Milano, Italy; 5 Department of Clinical Sciences and Community HealthLaboratory of Toxicology, University of Milan and Fondazione IRCCS Ca’ Granda, Ospedale Maggiore Policlinico, Milan, Italy

**Keywords:** metyrapone, medical treatment, Cushing’s syndrome, cortisol

## Abstract

**Objective:**

Metyrapone has been approved for the treatment of patients with Cushing’s syndrome (CS), but only few retrospective clinical studies are available. The aim of our study was the prospective assessment of metyrapone as pre-operative treatment.

**Design and methods:**

Before adrenalectomy, seven patients with ACTH-independent CS due to adrenal adenoma were prospectively treated with metyrapone for 3 months in three tertiary academic centers, with endocrine work-up and clinical evaluation at screening and at predefined evaluation time points (Days 14, 31, 48, 65, 82).

**Results:**

In all patients, UFC levels decreased up to normal range from baseline to Day 82 (609 (188–1476) vs 69 (28–152) nmol/24 h, *P* < 0.02), with a reduction of serum and salivary cortisol levels, and no significant increase of plasma ACTH and serum DHEAS levels. Clinical improvement was reported on quality of life (+16.7 (+4.2; +52.00) points, *P* < 0.04) and pressure control (systolic pressure, −25 (−52; −10) mmHg, *P* < 0.01; diastolic pressure, −16 (−50; +2 mmHg), *P* < 0.03). No significant change in weight, electrolytes, glycemic and lipid profile was reported. Although in women a significant increase of testosterone and androstenedione was reported, no worsening of clinical hyperandrogenism was observed. All drug-related adverse events (nausea, fatigue, low grade fever, edema of lower limbs and facial rash) were grade 1 or 2 and generally transient.

**Conclusions:**

This prospective pilot study demonstrated that metyrapone is effective in normalizing biochemical and clinical parameters in patients with CS due to adrenal adenoma before surgical intervention, with minimal side effects.

## Introduction

Cushing’s syndrome (CS) is a condition of endogenous hypercortisolism caused by different pathological entities that is classified in two variants: (i) ACTH-dependent CS, due to a corticotroph pituitary adenoma or an ectopic tumor; (ii) ACTH-independent CS, due to a primary adrenal process (tumor or hyperplasia). Whatever the cause, CS is associated with a huge burden of metabolic complications (hyperglycemia, dyslipidemia), cardiovascular and cerebrovascular diseases (hypertension, coronary artery disease, heart failure, stroke, thromboembolic events), increased susceptibility to infections and mood disorders. Therefore, CS is characterized by impaired quality of life (1) and increased mortality, with a reported standard mortality ratio between 2.0 and 4.0 (2). Because of these clinical consequences, CS requires a prompt and definitive resolution since the duration of exposure to cortisol excess is a factor portending a worse prognosis (3). Surgery is the mainstay of treatment. If surgery fails, or is unfeasible, medical treatment can temporarily control excessive cortisol production and ameliorate its clinical manifestations.

The inhibitors of steroidogenesis are the most used drugs to suppress cortisol secretion (4). In particular, metyrapone, which affects cortisol secretion by inhibiting the 11β-hydroxylase enzyme (CYP11B1) (5, 6), has a solid reputation of efficacy and entered clinical use since his introduction in 1958 (7); however, the drug has never been studied prospectively. Retrospective studies have shown that metyrapone is effective in promptly reducing cortisol levels. Cortisol dropped within target levels after 48–72 h from initiation of treatment with no later escape (8, 9). Adverse effects associated to treatment were either directly related to the drug intake (nausea, vomiting, dizziness, sedation, headache) or secondary to the metyrapone-induced alteration of steroid synthesis (hirsutism, hypertension) (8, 9).

In patients with ACTH-dependent CS, the block of CYP11B1 results in a compensatory rise of pituitary ACTH secretion that may lead to accumulation of steroid precursors with weak mineralocorticoid activity, potentially producing increased blood pressure, edema and hypokalemia. Moreover, the treatment leads to a shift of steroidogenesis toward the androgen pathway that may result in hirsutism and acne in women. However, in ACTH-independent CS, the compensatory rise in ACTH might not occur because ACTH secretion is chronically suppressed by autonomous cortisol secretion, and this may ameliorate the safety profile.

We hypothesized that metyrapone could be a perfectly suitable drug for a preoperative treatment of patients with ACTH-independent CS, since the fast action of the drug may allow a prompt improvement of clinical features of CS with a presumably favorable safety profile due to suppressed ACTH secretion in such patients. Therefore, we designed a prospective open-label pilot study of a short course of metyrapone treatment to improve patient’s conditions and quality of life in preparation to adrenalectomy.

## Subjects and methods

### Patients

**Figure 1:**
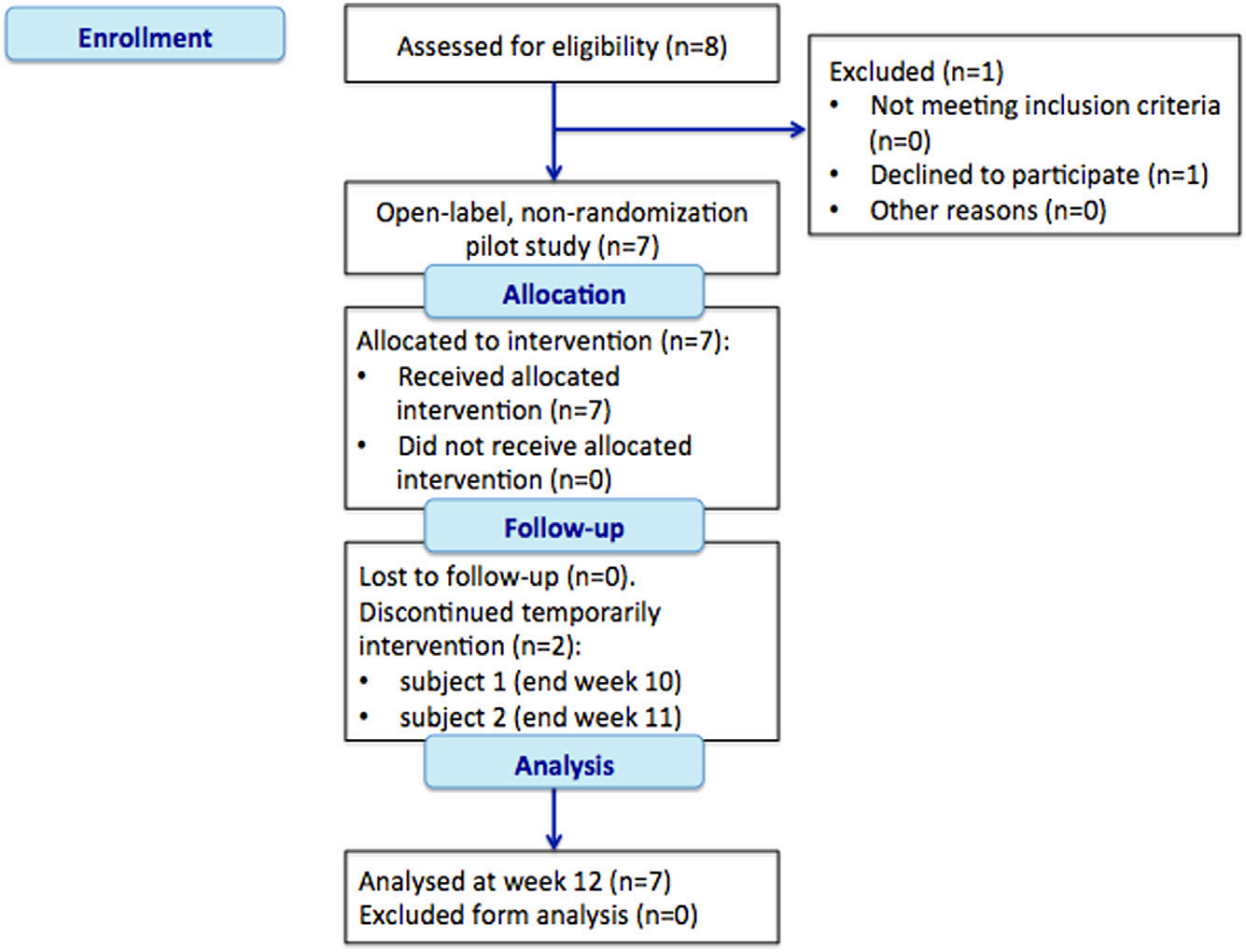
Flow chart indicating the different phases of the study to week four as recommended by the TREND statement (10). Subjects 1 and 2 discontinued treatment for a few days due to nausea, fatigue and low-grade fever.

For the purpose of this study, we enrolled prospectively seven consecutive patients with ACTH-independent CS due to an adrenal adenoma scheduled for adrenalectomy, who were referred to three tertiary academic centers (San Luigi Gonzaga Hospital – University of Turin; University Hospital of Padova; Sant’Andrea Hospital – Sapienza University of Rome), between March 2014 and February 2016. All patients presented with a clear phenotype of CS and none of the tumors was detected serendipitously. They were two men and five women, aged 30–66 years (median 40 years). Inclusion criteria for the study were age ≥18 years, confirmed diagnosis of ACTH-independent CS (validated by all the following criteria: (i) two 24-h urinary collections for urinary free cortisol (UFC) both >1.5 times the upper normal value, within 2 weeks prior to enrollment; (ii) ACTH plasma levels lower than the normal range; (iii) serum cortisol following a 1 mg dexamethasone suppression test (DST) >138 nmol/L (5 µg/dL); (iv) monolateral adrenal tumor with unequivocal radiological features of benignity on an unenhanced CT scan performed within 4 weeks prior to enrollment (size <4 cm, regular shape and margins, homogeneous density <10 HU); ECOG Performance Status ≤2. Effective contraception was required, but hormonal contraceptives were not allowed. Exclusion criteria were prior treatment with metyrapone or any drug specifically directed against cortisol excess, adrenal tumor of uncertain origin (suspicious for malignancy), known hereditary syndrome (Carney’s syndrome, McCune–Albright syndrome, MEN-1), any severe concomitant disease or impairment of organ function that may prevent the participation in the study procedures, pregnancy, breast-feeding, history of alcohol or drug abuse, history of recent or active malignancy, acute or chronic uncontrolled infections, non-collaborating patient. The study was designed in agreement with the Declaration of Helsinki and was approved by the Ethics Committee of San Luigi Gonzaga Hospital (‘Comitato Etico Interaziendale A.O.U. San Luigi Gonzaga’). The clinical trial was registered in the European Clinical Trial database (EudraCT number: 2013-002063-26). The patients volunteered for the study and gave their written informed consent. The study is reported according to the Transparent Reporting of Evaluations with Nonrandomized Designs (TREND) (10). The flow chart in Fig. 1, as recommended by the TREND statement, describes the different phases and design of the study.

### Study objectives

The aim of our study was the prospective assessment of metyrapone as pre-operative treatment.

#### Primary objective

The primary objective of this study is to assess the efficacy of metyrapone in attaining normalization of 24-h urinary free cortisol (UFC) excretion or a ≥50% decrease from baseline using the mean of 2 UFC measurements within 3 months of treatment.

#### Secondary objectives

- Time to response.
- Dose–response relationship.
- Effect of metyrapone on levels of serum cortisol, UFC, salivary cortisol, ACTH, 11-deoxycortisol, total testosterone, androstenedione, DHEAS in terms of percent variation relative to baseline.
- Improvement of the clinical signs associated to hypercortisolism (blood pressure, BMI, waist).
- Improvement of quality of life, assessed by the CS questionnaire.
- Improvement of the metabolic alterations associated to hypercortisolism (fasting hyperglycemia, dyslipidemia).
- Safety and tolerability of oral assumption of metyrapone at different doses.

### Study protocol

The study was done in an outpatient setting at three centers involved in patient recruitment. It consisted in two periods: a screening period (up to 2 weeks, from Day −14 to Day 0 screening of potentially eligible patients) and a treatment period (82 ± 8 days, with scheduled visits at Day 7, 14, 31, 48, 65 and 82). The study period included 24 months of enrolling period (from March 2014 to February 2016) and 3 months of treatment period (last scheduled visits in May 2016).

During treatment period, all patients received from investigators the same oral metyrapone formulation (metyrapone 250 mg, tablets, provided by HRA Pharma, Paris, France), at the initial dose of 250 mg thrice daily with further titration (up or down-titration) at scheduled times on individual basis according to hormone levels, clinical features and patient’s tolerance. Compliance for study drug has been assessed by counting capsules at the scheduled patient visits. Patients have been asked to record the date and time of dosing and number of study drug capsules taken. Adherence to the study protocol will be reinforced at each visit and during the weekly contact (telephone or email).

The metyrapone efficacy was monitored by UFC levels. The current dose of metyrapone should have been continued in case of normalization of UFC. If UFC was not normalized, the dose should have been up titrated by 250 mg per day, at each scheduled visit. The possible development of hypoadrenalism was monitored by the evaluation of serum cortisol levels. In case of cortisol levels (before metyrapone intake) <248 nmol/L (<9 µg/dL), metyrapone should have been reduced by at least 250 mg.

A complete physical examination (including general appearance, HEENT (head, eyes, ears, nose, throat), as well as dermatologic, cardiovascular, respiratory, gastrointestinal, extremities/musculoskeletal and neurologic body system) was performed at screening and at each scheduled visit. Vital signs including blood pressure, heart rate, respiratory rate and oral body temperature have been collected. Weight and waist circumference have been measured at baseline and during each visit with the same scales and tape measures, respectively, to ensure consistency. Twelve-lead ECG tracings have been obtained in triplicate from all patients. Blood samples for biochemical test (glycemic and lipid profile, electrolytes) were obtained at screening and at each visit. For hypertensive and diabetic patients, number of specific drugs and daily doses were recorded. An endocrine work-up was required at screening and at predefined evaluation points (Days 14, 31, 48, 65, 82). Blood samples for hormone measurement (serum cortisol, ACTH, total testosterone, androstenedione, DHEAS, 11-deoxycortisol) were obtained between 08:00 h and 09:00 h before the morning metyrapone dose, while UFC excretion was measured on a daily urine collection (UFC reference range <165 nmol/24 h, corresponding to the 95th percentile value of a reference population of normal subjects). Salivary cortisol was evaluated collecting salivary samples thrice daily at 08:00, 16:00, 23:00 h, immediately before each metyrapone dose, by chewing a cylindrical cotton swab (Salivette, Sarstedt, Nümbrecht, Germany) for about 2 min. The subjects were told not to eat and brush their teeth since at least 3 h before the collection. Samples were centrifuged at 1500 ***g*** for 3 min, the cotton swab was removed, and the collected saliva samples were frozen at −20°C until assayed.

Hormone measurements were centralized in a reference laboratory (Laboratorio di tossicologia, Fondazione IRCCS Ca’ Granda, Ospedale Maggiore Policlinico Milano). Results of serum and urinary cortisol measurements were available within 3 days to allow for prompt dose titration of metyrapone.

The performance status of all patients was graded according to the ECOG Performance Status scale and the toxicity associated with study drug was rated using the NCI CTCAE v 4.0 (11). Presence of symptoms of cortisol excess was annotated and judged by the investigator by using the specific Cushing questionnaire (12). Patients were steadily monitored throughout the study for the occurrence of adverse events. All adverse events occurring from the beginning of the study and up to 30 days after the end of the administration of the drug were recorded, regardless of the potential relationship to the study drug. The date of onset, severity, seriousness and investigator’s opinion of potential relationship of the event to study drug were recorded.

### Hormone measurements

#### Steroids in serum

To analyze cortisol, 11-deoxycortisol, DHEAS, testosterone and androstenedione levels by LC–MS/MS, an IVD-MS steroids serum kit (MassChrom, Steroids in Serum/Plasma, Chromsystems, Gräfelfing, Germany) was used. Chromatographic separation and mass spectrometry of the samples was performed with a high-performance liquid chromatography (HPLC, Shimadzu, Milano, Italy) interfaced with a Sciex 4500 MD mass spectrometer (Sciex, Milano, Italy). Samples were prepared according to the manufacturer’s instructions.

#### Salivary and urinary cortisol

The determination of both salivary and urinary cortisol was carried out in the presence of cortisol-d4 as deuterated internal standard, by LC–MS/MS after submitting the saliva and urine sample to a purification by an online TurboFlow system (Thermo Scientific), using a Cyclone column (50 mm length, 0.5 mm internal diameter, Thermo Scientific), as previously described (13). Cortisol was separated in liquid chromatography using a C18 reversed-phase column (Hypersil Gold, 50 mm length, 2.1 mm internal diameter, 3 µm particle size, Thermo Scientific), with a gradient of aqueous ammonium formate (5 mM) with 0.1% formic acid and methanol as eluent, flowing at 0.7 mL/min. Detection and quantification were performed by a triple quadrupole mass spectrometer (TSQ Quantum Access, Thermo Scientific) equipped with a heated-electro spray ionization source (H-ESI), operating in the positive ion mode. The method for detecting salivary cortisol had a precision, assessed as percent coefficient of variation, of less than 5%, accuracy between 99 and 102% and a limit of quantification (LOQ) of 1 nmol/L. The throughput was about 100 samples/day. The method for detecting UFC had a precision, assessed as percent coefficient of variation, of less than 10%, accuracy between 98 and 107% and a limit of quantification (LOQ) of 3 nmol/L.

#### Plasma ACTH levels

Plasma ACTH levels (pmol/L) were measured by chemiluminescent immunometric assay (Immulite 2000, Siemens Medical Solutions Diagnostics) with an inter-assay coefficient of variation ranged from 6.1 to 10.0%, an intra-assay coefficient of variation ranged from 6.7 and 9.5% and sensitivity of 1 pmol/L.

### Statistical analysis

This is a pilot study and no formal power calculation has been performed.

This study is exploratory in nature. All statistical analyses were performed as intention to treat with descriptive and exploratory purposes and the results of statistical tests presented with CIs. Data were collected by means of an eCRF and centralized to the Unit of San Luigi Hospital for further processing. All statistical analyses were performed with Statistica (StatSoft) statistical software. The Mann–Whitney non-parametric test will be used for analysis of continuous variables, the chi-square test will be used for nominal or *ordinal* explanatory and response *variables*. Correlation analyses were determined by calculating the Spearman’s R coefficient. *P* values of less than 0.05 were considered to indicate statistical significance. Rates and proportions were calculated for categorical data and medians and ranges for continuous data.

## Results

**Figure 2:**
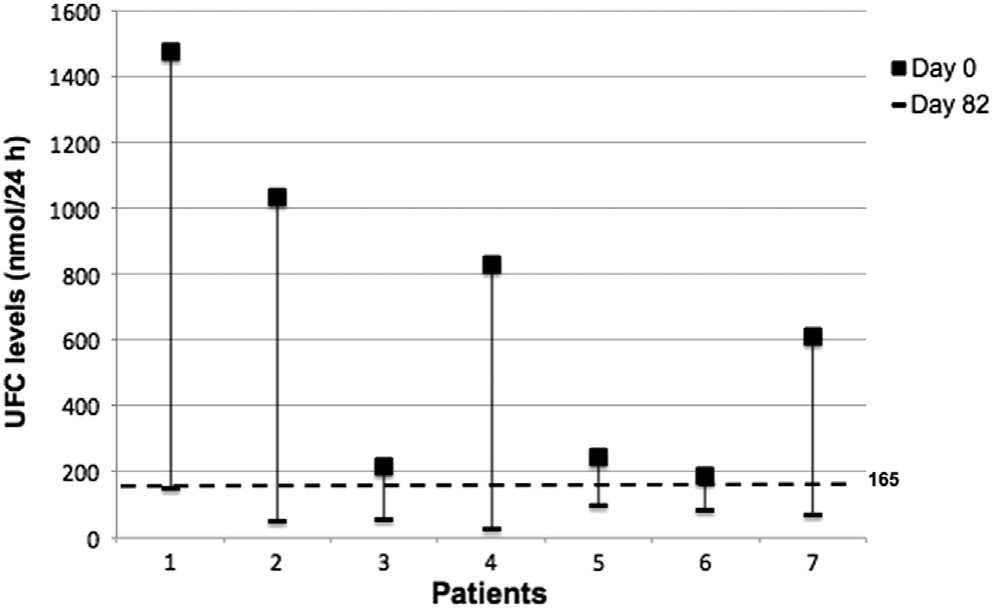
UFC levels at Day 0 and Day 82 in seven patients treated with metyrapone. 165 nmol/24 h is the upper limit of the reference range.

Baseline characteristics of the patients are provided in Table 1. At baseline, 4/7 patients had mild-to-moderate hypercortisolism and the remaining had severe hypercortisolism. All patients completed the treatment period and the median daily doses of metyrapone were as follows: 750 mg at Day 14, 750 mg at Day 31 (range 500–1250 mg), 1000 mg at Day 48 (250–1750 mg), 750 mg at Day 65 (250–1750 mg) and 750 mg at Day 82 (250–1750 mg). At baseline, UFC levels were 609 nmol/24 h (188–1476 nmol/24 h) and they decreased in all patients from Day 0 to Day 82 (Fig. 2). In the overall group, the median percentage change in UFC level comparing to baseline was −76.0% (range −88.4% to +55.3%; *P* = 0.05) after 2 weeks and −84.5% (range −97.8% to +31.9%; *P* < 0.03) after 1 month of treatment, respectively. Normalization of UFC level was reported in all patients at Day 65 (85 nmol/24 h, range 36–128 nmol/24 h), and Day 82 (69 nmol/24 h, range 28–152 nmol/24 h), with median percentage change comparing to baseline of −90.1% (range −94.1% to −46.7%; *P* < 0.02) and −88.7% (range −96.6% to −56.6%; *P* < 0.02), respectively. Also serum and salivary cortisol levels progressively decreased from Day 0 to Day 82. At the end of the treatment period, the median percentage change in morning serum cortisol was −35.2% (range −73.5% to +9.7%; *P* < 0.05), while the median percentage change in salivary cortisol was −63.0% (range −73.2% to +8.6%; *P* = 0.06) at 08:00 h, −66.9% (range −83.9% to −13.6%; *P* < 0.04) at 16:00 h, and −79.8% (range −91.2% to −55.2%; *P* < 0.02) at 23:00 h, respectively (Table 2). During the treatment period, median plasma ACTH and serum DHEAS did not increase significantly (Table 2). Serum testosterone and serum androstenedione levels significantly increased in women, although values were in the normal range (Table 2). In all patients serum 11-β-deoxycortisol levels significantly increased from Day 0 to Day 82 (Table 2). During the treatment, an improvement in the quality of life and pressure was observed (Table 3). The median changes from Day 0 to Day 82 were as follows: health-related quality of life score, +16.7 points (range +4.2 to +52.00, *P* < 0.04); systolic blood pressure, −25 mmHg (range −52 to −10 mmHg, *P* < 0.01); diastolic blood pressure, −16 mmHg (range −50 to +2 mmHg, *P* < 0.03). Moreover, 3/7 patients were able to reduce the number of anti-hypertensive drugs. No significant change in weight, waist circumference, glycemic profile and lipid profile was reported (Table 3). During treatment, sodium and potassium levels were in the normal range.

**Table 1:** Baseline characteristics of patients.

Characteristic	Patients (*n* = 7)
Age (years)	
Median	40
Range	30–66
Sex (*N*)	
Men	2
Women	5
Time since diagnosis (months)	
Median	2
Range	1–7
Size of adenoma (mm)	
Median	30
Range	24–31
Hypertension (*N*)	
Yes	5
No	2
Diabetes mellitus (*N*)	
Yes	1
No	6
Severity of hypercortisolism (*N*)	
Mild	2
Moderate	2
Severe	3

Mild hypercortisolism = UFC level <2 times the upper limit of the normal range (ULN); moderate hypercortisolism = UFC level between 2 and 5 times the ULN; severe hypercortisolism = UFC level >5 times the ULN.

**Table 2:** Hormonal characteristics of patients.

Characteristic	Day 0	Day 82	*P* Value
UFC (nmol/24 h)			
Median (range)	609 (188–1476)	69 (28–152)	**0.017**
Reference range	12–165		
Serum morning cortisol (nmol/L)			
Median (range)	599 (392–665)	306 (174–715)	**0.047**
Reference range	138–690		
Salivary cortisol h 08:00 (nmol/L)			
Median (range)	21 (6–39)	6 (6–14)	0.062
Reference range	1–15		
Salivary cortisol h 16:00 (nmol/L)			
Median (range)	16 (3–36)	4 (2–15)	**0.032**
Reference range	1–5		
Salivary cortisol h 23:00 (nmol/L)			
Median (range)	14 (5–32)	2 (1–14)	**0.016**
Reference range	1–3		
ACTH (pmol/L)			
Median (range)	1 (1–2)	1 (1–11)	0.42
Reference range	1–10		
Testosterone in men (nmol/L)			
Median (range)	3 (3–4)	8 (4–12)	0.43
Reference range	9–35		
Testosterone in women (nmol/L)			
Median (range)	1	2 (1–3)	**0.033**
Reference range	1–3		
Androstenedione in men (nmol/L)			
Median (range)	1	2 (1–2)	0.13
Reference range	2–9		
Androstenedione in women (nmol/L)			
Median (range)	1 (1–10)	7 (3–12)	**0.014**
Reference range	2–9		
11-β-Deoxycortisol (nmol/L)			
Median (range)	2 (1–4)	69 (4–92)	**0.017**
Reference range	1–9		
DHEAS (µmol/L)			
Median (range)	1 (1–8)	1 (1–8)	0.44
Reference range	<17 (M)<14 (childbearing age)<3 (menopausal age)		

For all variables, there were seven valid cases. Bold text indicates statistically significant values.

**Table 3:** Clinical and biochemical characteristics of patients.

Characteristic	Day 0	Day 82	*P* Value
Glycemia (mmol/L)			
Median (range)	5.6 (4.4–8.2)	4.9 (4.4–6.1)	0.15
Reference range	4.0–5.5		
HOMA index			
Median (range)	2.7 (2.2–8.8)	2.7 (1.1–3.9)	0.27
Total cholesterol (mmol/L)			
Median (range)	5.7 (4.4–5.8)	5.2 (4.2–6.1)	0.22
Reference range	<5.2		
Triglycerides (mmol/L)			
Median (range)	1.2 (0.4–2.9)	1.3 (0.4–2.6)	0.89
Reference range	<1.7		
BMI (kg/m^2^)			
Median (range)	27.1 (23.3–35.8)	26.1 (22.8–36.9)	1.00
Waist circumference (cm)			
Median (range)	90 (88–125)	91 (87–126)	0.88
HrQoL score			
Median (range)	39.8 (22.9–68.7)	65.6 (27.1–92)	**<0.03**
SBP (mmHg)			
Median (range)	140 (120–180)	128 (110–130)	**<0.01**
DBP (mmHg)			
Median (range)	96 (85–124)	80 (70–98)	**<0.03**
Sodium (mmol/L)			
Median (range)	139 (136–142)	139 (138–145)	0.46
Reference range	136–145		
Potassium (mmol/L)			
Median (range)	4.0 (3.1–4.8)	4.1 (3.8–4.4)	0.46
Reference range	3.6–5.2		

For all variables, there were seven valid cases. Bold text indicates statistically significant values.

BMI, body mass index; DBP, diastolic blood pressure; HRQoL, health-related quality of life; SBP, systolic blood pressure.

There were no deaths or serious adverse events during treatment and metyrapone was well tolerated by all subjects. All drug-related adverse events were grade 1 or 2, and generally transient. The reported drug-related effects include nausea, fatigue, low grade fever (in 2/7 patients), which resolved after discontinuing treatment for a few days and re-introducing a lower dose (250 mg less); lower limb edema (2/7 patients), which resolved with introduction of 50–100 mg spironolactone; transient facial rash (in 2/7 patients). Hirsutism and acne were not reported in female patients, and in one case, an improvement of hirsutism and menstrual periodicity was reported.

## Discussion

We did a prospective, open-label, pilot study involving a pre-operative treatment with metyrapone in seven patients with ACTH-independent CS due to adrenal adenoma scheduled for adrenalectomy. The rationale of this study is that patients with adrenal adenoma should be the best candidates to benefit from metyrapone because they may lack a compensatory rise of ACTH secretion during treatment, since ACTH is chronically suppressed by autonomous cortisol secretion. This could ameliorate the safety profile of metyrapone, avoiding side effects caused by an ACTH-mediated increase of steroid precursors with weak mineralocorticoid activity (increased blood pressure, edema and hypokalemia) or androgen effects (hirsutism, acne, irregular menstrual cycles). The medical need of a pre-operative course of metyrapone is in the possibility to control a severe Cushing, thus reducing associated morbidity that increases operative risks and complications. Moreover, an effective medical treatment of cortisol excess that provides useful palliation of Cushing stigmata, thus improving clinical conditions, quality of life and avoiding incoming complications, is useful even in mild-moderate Cushing whether a long time lag between diagnosis and surgery is expected. As a matter of fact, patients with CS should be referred to high-volume expert surgical centers, which in Italy have usually long waiting lists for benign conditions.

Whether it is known that metyrapone may control rapidly cortisol excess in 50–75% of patients with CS due to different causes (4), only data collected retrospectively are available to prove the efficacy of metyrapone. This is to our knowledge the first prospective study on metyrapone use in CS. Moreover, there is limited available information on the outcome of metyrapone treatment in patients with ACTH-independent CS. Verhelst and colleagues (8) observed an effective reduction of serum cortisol levels in 13/16 patients with cortisol-secreting adrenal tumors (10 adrenal adenoma, 6 adrenocortical carcinoma) treated with a median daily dose of 1750 mg (range 750–6000). Daniel and colleagues (9) reported 124 patients treated with metyrapone monotherapy before any surgical intervention, including 25 benign adrenal disease and 7 adrenocortical carcinoma (ACC), for an average of 4.0 months. In this group, control of daily cortisol levels was achieved in 50% of patients with available data, while control of UFC levels was found in 35% of patients with available data. The median daily start dose of metyrapone was 750 mg (range 500–2250) in patients with benign adrenal disease, and 1500 mg (750–2000) in patients with ACC, while the median daily final dose was 1000 mg (500–4000) and 1000 mg (750–1500), respectively.

Our findings demonstrate a prompt and effective activity of metyrapone also in patients with severe hypercortisolism, who showed a UFC reduction greater than 80% after 2 weeks of treatment. Most of our patients (5/7) achieved UFC normalization after 1 month of treatment and the normalization of UFC was found in all patients at the end of the treatment period (Day 82). The median final dose of metyrapone in our study was of 750 mg daily (range 250–1500 mg), lower than that in other reported series (8, 9). This is likely due to differences in study design (retrospective series vs a prospective study) and included patients (patients with benign adrenal disease or ACC in previous series vs patients with adrenal adenoma in our study).

Normalization of UFC was accompanied by reduction in serum and salivary cortisol, and marked clinical improvement. Scores for health-related quality of life increased, and we observed a significant drop in systolic and diastolic blood pressure. The prompt and remarkable effect on blood pressure outlines the tight link between hypercortisolism and hypertension (14, 15, 16, 17, 18, 19, 20). Improvement in blood pressure occurred despite the expected rise of 11-desoxycortisol, a cortisol precursor with weak mineralocorticoid activity, due to the metyrapone-induced inhibition of the CYP11B1 (21, 22, 23). However, this might have contributed to lower limb edema in two patients, which resolved with 50–100 mg of spironolactone.

Interestingly, we did not observe a compensatory increase in ACTH secretion (21); ACTH remained below limits of detection in four patients and increased into the normal range in the remainders. Moreover, we find only a moderate increase in androgens with no clinical effect; conversely, a female patient reported amelioration of hirsutism and menstrual periodicity, probably due to the beneficial effect of cortisol reduction. This would confirm our hypothesis that the lack of ACTH rise due to the chronic pituitary suppression in patients with ACTH-independent CS limited the shift toward the androgen pathway. Verhelst *et al*. in a retrospective study evaluated ACTH patterns in patients with adrenal-dependent CS on metyrapone and did not find any significant ACTH increase in ten patients with adrenal adenoma and six patients with adrenal carcinoma (8). Instead ACTH levels were not reported by Daniel *et al*. (9).

The post-operative follow-up was heterogeneous among the different centers and we have incomplete information. After adrenalectomy, all patients received glucocorticoid replacement therapy (cortisone acetate). At 6–12 months after adrenalectomy, all patients had normal blood pressure (median systolic blood pressure, 115 mmHg, 105–120; median diastolic blood pressure, 80 mmHg, 70–85).

To summarize, we have demonstrated for the first time the activity of metyrapone in a prospective series of patients with adrenal-dependent CS. We are aware of the limitations of our study due to the small number of patients, but we think that our results support the feasibility of a short-lived treatment with metyrapone before adrenal surgery. We have shown that metyrapone is able to normalize cortisol excess rapidly, thus leading to a remarkable improvement of patient conditions, and this makes the drug perfectly suitable for a pre-operative use. We have also shown that a short-lived treatment has a very favorable safety profile in patients with adrenal-dependent CS, due to either the condition of ACTH independency or the limited duration of treatment. Since our patient characteristics represent the average patient with overt CS due to an adrenal adenoma, we think that our results are generalizable to this patient population.

## Declaration of interest

Massimo Terzolo has received research grant from HRA and Novartis; the others authors have stated explicitly that there are no conflicts of interest in connection with this article. Massimo Terzolo is an editor of the journal.

## Funding

This research did not receive any specific grant from any funding agency in the public, commercial or not-for-profit sector.
